# Inflammatory Myofibroblastic Tumor of the Prostate Gland: A Case Report From North-Central Nigeria

**DOI:** 10.7759/cureus.82556

**Published:** 2025-04-19

**Authors:** Kevin N Ezike, Ijeoma A Okwudire-Ejeh, Bamnan C Dallang, Emmanuel O Adugba, Ugonna N Ezike

**Affiliations:** 1 Anatomic Pathology and Forensic Medicine, Nile University of Nigeria, Abuja, NGA; 2 Anatomic Pathology and Forensic Medicine, Asokoro District Hospital, Abuja, NGA; 3 Urology, Asokoro District Hospital, Abuja, NGA; 4 General Practice, Kubwa General Hospital, Abuja, NGA

**Keywords:** alk gene translocation, bladder outlet obstruction, inflammatory myofibroblastic tumor, nigeria, prostate gland

## Abstract

Inflammatory myofibroblastic tumors (IMTs) are mesenchyme-derived tumors of undetermined malignant potential, generally considered low-grade tumors. They occur within various organs, with the abdominal cavity as the most common site of occurrence. The male genitourinary system, especially the prostate gland, is among the least common sites of occurrence. IMTs are generally diagnosed in the young, where they present as circumscribed masses with symptoms referable to the location and size. Occasionally, however, they present in the elderly. The etiopathogenesis is postulated to be related to an abnormality in chromosome 2p23.5, and association with anaplastic lymphoma receptor tyrosine kinase (ALK) positivity on immunohistochemistry (IHC) has therapeutic and prognostic implications. Distinguishing IMTs from non-neoplastic mimics like inflammatory pseudotumor (IPT) is important because they have similar microscopic features, characterized by benign spindle and stellate cells of variable cellularity, admixed with mixed inflammatory cell infiltrates in a fibrocollagenous background. Surgery is the treatment of choice, with a favorable outcome in most cases. Targeted therapy with the tyrosine kinase inhibitor (TKI) crizotinib is increasingly touted in recurrent cases or those not amenable to surgery.

We present a case of IMT in a 77-year-old Nigerian male who was clinically diagnosed as a case of benign prostatic hyperplasia (BPH), in order to highlight the imperative for a high index of suspicion in the histopathological evaluation of prostatic lesions.

## Introduction

Inflammatory myofibroblastic tumors (IMTs) are mesenchymal neoplasms of intermediate malignant potential that can occur in any part of the body [1,2]. They are generally regarded as tumors of children and young adults, and rarely occur in the elderly [2]. Histologically, they are composed of spindle cells with variable cellularity and growth patterns, admixed with inflammatory cells - particularly lymphocytes, plasma cells, and eosinophils [1,2].

IMTs are rare, with 75% of cases occurring in the abdominal cavity, especially in the mesentery, greater omentum, and retroperitoneal space, and less commonly in the head and neck, lungs, urinary bladder, central nervous system, and the female genital tract [3]. Their clinical features are site-dependent, and in the rare occasions in which they occur in the prostate gland, they may present with hematuria and dysuria [3,4]. IMTs typically pose a diagnostic conundrum for pathologists when they occur in the prostate gland because the samples are predominantly encountered initially as small core biopsies [4].

The etiology and pathogenesis of IMTs are unclear. However, a number of risk factors have been associated, such as trauma, smoking, and IgG4-related infections, including viral infections [5-7]. Up to 80% of these tumors exhibit kinase fusion as the molecular abnormality, with abnormalities in chromosome 2p23.5, and approximately 60% are anaplastic lymphoma receptor tyrosine kinase (ALK) positive on immunohistochemistry (IHC) [1,5].

We present a case of IMT of the prostate gland in a 77-year-old male, which we believe to be the first such report in a Nigerian, initially diagnosed clinically as benign prostatic hyperplasia (BPH).

## Case presentation

A 77-year-old male, Nigerian, presented at a private medical facility in Abuja, Nigeria, with the sudden onset of inability to pass urine and severe lower abdominal pain. He had a prior history of difficulty in passing urine of more than one year's duration, for which he had been investigated and was on conservative management for suspected BPH. There was an associated history of recent hematuria and weight loss.

Physical examination revealed moderate pallor, blood pressure of 120/70 mmHg, and suprapubic tenderness, and digital rectal examination confirmed a moderately enlarged prostate gland with smooth contours and transmitted tenderness. A two-way urethral catheter was inserted, which immediately relieved the obstruction and his symptoms but was observed to be draining bloody urine. A working diagnosis of acute bladder outlet obstruction due to BPH, with a need to rule out carcinoma of the prostate, was made.

The results of his preliminary laboratory investigations were as follows: serum prostate-specific antigen (PSA), 12.4 ng/mL; packed cell volume (PCV), 26%; random blood glucose (RBG), 6.9 mmol/L; serum electrolytes: sodium (Na^+^), 136.0 mmol/L; potassium (K^+^), 5.7 mmol/L; chloride (Cl^-^), 99.0 mmol/L; urea, 91.5 mmol/L; and creatinine, 150.2 mmol/L.

A few hours following the presentation, the two-way urethral catheter stopped draining (blood clots were observed within the catheter), with increasing lower abdominal pain and suprapubic tenderness. Intramuscular pentazocine was given for pain relief. A three-way urethral catheter was then inserted, which enabled continuous bladder irrigation and relief of the obstruction. A transrectal ultrasound (TRUS)-guided biopsy of the prostate was then scheduled, but due to persistent bleeding and a deteriorating circulatory system, he had an emergency simple (open) prostatectomy done on the day following presentation. One unit of blood was transfused preoperatively, and three units postoperatively over a three-day period. His immediate postoperative condition was stable. Postoperative laboratory investigations showed PCV (27%) and electrolytes and urea (Na^+^, 138.1 mmol/L; K^+^, 3.6 mmol/L; Cl^-^, 98.7 mmol/L; urea, 87.8 mmol/L; and creatinine, 105.6 mmol/L). The prostatectomy specimen was preserved in 10% neutral buffered formalin and sent for histopathological evaluation.

**Figure 1:**
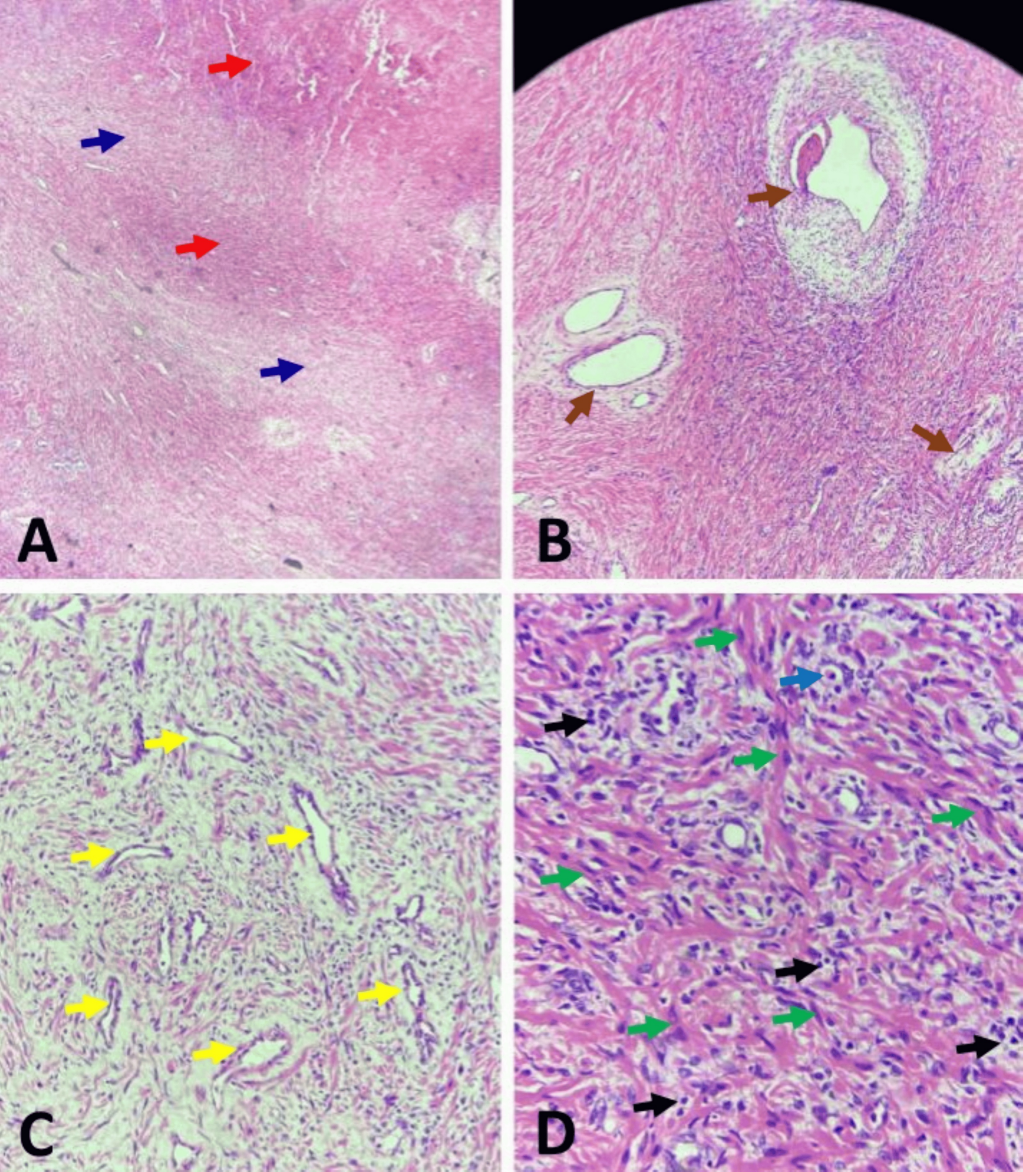
Inflammatory myofibroblastic tumor of the prostate gland (A) Note haphazard fascicles of spindled to stellate cells, exhibiting variable areas of hypo- and hypercellularity (blue and red arrows, respectively), set in vascularized fibrocollagenous stroma (H&E, x40). (B) Note prominent vasculature comprising medium- to large-sized, thick-walled vascular channels (brown arrows) in a hypercellular area (H&E, x100). (C) Note prominent vasculature comprising small- to medium-sized, thin-walled vascular channels (yellow arrows) in a hypocellular area (H&E, x100). (D) Note short fascicles of spindle cells with eosinophilic fusiform cytoplasm and slender, often vesicular, bland nuclei (green arrows), admixed with moderate infiltrates of lymphocytes and plasma cells (black arrows), occasional eosinophils (blue arrow), and fibrocollagenous stroma (H&E, x400). H&E: hematoxylin and eosin

**Figure 2:**
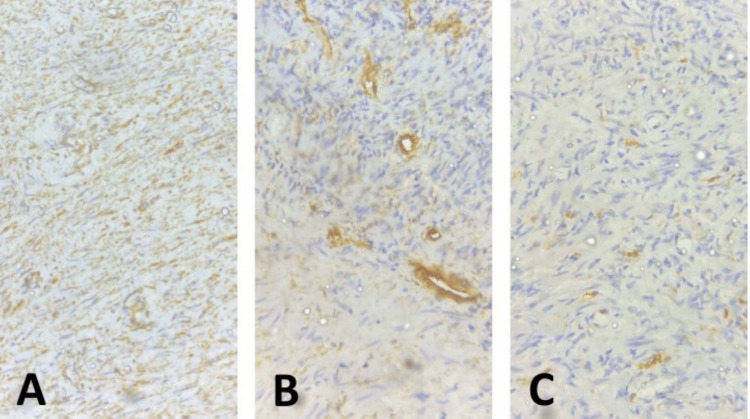
IHC profile of inflammatory myofibroblastic tumor of the prostate gland (A) Diffusely positive SMA stain: note intense and diffuse cytoplasmic staining (golden brown coloration) of lesional cells (SMA IHC, x400). (B) Negative CD34 stain: note lack of staining of lesional cells and intense membranous staining (golden brown discoloration) outlining vascular channels (CD34 IHC, x400). (C) Negative S100 stain: note lack of staining of lesional cells (S100 IHC, x400). IHC: immunohistochemistry; SMA: smooth muscle actin

The pathological examination revealed gross findings of a circumscribed greyish-white mass measuring 5.5 x 4.2 x 3 cm and weighing 42 g. Serial sectioning showed a solid, greyish-white surface with a vague whorled appearance. Microscopic sections showed a circumscribed mesenchymal proliferation composed of haphazard fascicles of spindled to stellate cells, exhibiting variable areas of hypo- and hypercellularity, set in markedly fibrocollagenous stroma, and admixed with moderate mixed inflammatory infiltrates, predominantly lymphocytes and plasma cells, with occasional eosinophils. The spindle cells had eosinophilic, fusiform cytoplasm and variably slender and plump central nuclei. No prostatic acini were seen. The vasculature was prominent, consisting of medium- to large-sized, thick-walled vessels, and frequent foci of congested, medium-sized, thin-walled vessels, reminiscent of granulation tissue-type vasculature. There was no cytological atypia or significant mitotic activity. IHC stains showed that the tumor cells were diffusely positive for smooth muscle actin (SMA) and negative for CD117, S100, and CD34, respectively. A diagnosis of IMT was made (Figures 1-2).

The patient’s post-operative management included continuous bladder irrigation with antibiotics and analgesic therapy. He was discharged on the ninth day post-surgery, following successful urethral catheter removal on day 7. He remained stable and symptom-free on both the seventh-day and one-month post-discharge reviews, respectively. The wound site was healed, with minimal scar tissue formation. He has since been lost to follow-up.

## Discussion

IMT is a mesenchymal neoplasm of soft tissue originating from fibroblastic and myofibroblastic cells [8]. It is often considered to be a borderline or low-grade malignant tumor [8,9]. IMTs have a long and storied history. They were first described in 1903 as inflammatory pseudotumor (IPT), but it was not until 1990 that they were categorized as a distinct entity, with their neoplastic nature established in 1999 [3].

The sites of occurrence are varied. Seventy-five percent of cases occur in the abdominal cavity, with a predilection for the mesentery, greater omentum, and retroperitoneal space [3]. Other sites include the head and neck, lungs, urinary bladder, prostate gland, central nervous system, and female genital tract [1,3]. While the urinary bladder is the most common site of occurrence in the male genital tract, occurrence in the prostate gland, as seen in our patient, is quite rare [8,10,11]. IMTs occur over a wide age range but occur preferentially in children and young adults, with children 10 years old and under showing a predilection for extrapulmonary tumors apart from the prostate gland [1,12,13]. Kim et al. reported a review of cases of IMTs of the prostate gland in the English literature that showed the mean age of occurrence to be 60.8 years [10].

The etiology of IMT is not always clear. An abnormal immune-inflammatory response to varied antigenic triggers has been postulated, and this may also explain the cytokine release responsible for up to a third of patients presenting with systemic symptoms, such as fever, night sweats, and weight loss [1,14]. Our patient’s complaint of weight loss may well be attributable to this hypothesis. The neoplastic nature of this lesion is buttressed by the presence of ALK rearrangement associated with the 2p23 locus, seen in 40%-60% of cases [10]. Although the ALK gene translocations identified in IMT are widely accepted as a neoplastic trigger, cases have been reported, including in the prostate gland, with negative ALK rearrangements [10,14,15].

Patients with prostatic tumors typically present with irritative or obstructive urinary symptoms, as well as hematuria and fever [10,12]. The presence of obstructive features and hematuria, as expected, would pose a diagnostic challenge vis-à-vis BPH and prostate cancer. Our patient’s symptomatology and presentation profoundly encapsulate this conundrum.

Morphologically, IMTs are typically grossly circumscribed solid masses with greyish white, variably whorled to rubbery and/or myxoid cut surfaces, and range in size from 1 cm to 20 cm [1,8]. Our patient’s tumor’s greyish white, faintly whorled appearance on cut section was the first pointer to a potential diagnosis other than BPH, thus highlighting the importance of vigilance and attention to detail in the gross examination of prostatectomy specimens. Microscopically, IMTs are characterized by the proliferation of myofibroblastic cells, admixed with inflammatory cells, including lymphocytes, plasma cells, and eosinophils [1]. Mitotic figures vary in number, and atypical forms are only infrequently seen [1,8]. The IHC profile of these tumors is nonspecific, showing positivity for such broad-spectrum panels as vimentin, SMA, and desmin, with occasional positivity for cytokeratins [1,8,11]. These markers are, however, insufficient for distinguishing IMTs from other spindle cell tumors, but the combination with known negative IHC stains like CD117, CD34, and S100 confers a higher degree of certainty [1,8]. ALK1 IHC positivity, when seen, is pathognomonic of IMT and has prognostic and therapeutic implications, as it opens the door for targeted therapy with the tyrosine kinase inhibitor (TKI), crizotinib [1,8,14]. Since ALK IHC was not available to us, we relied on the distinctive microscopic features of IMT on hematoxylin and eosin (H&E) and the combination of positive SMA and negative CD34, CD117, and S100 IHC stains to arrive at our patient’s diagnosis. In response to the variable rate of ALK expression assessed via IHC on the cell surface, recent authorship has recommended the use of next-generation sequencing (NGS) to further interrogate IMT cases that test negative for ALK1 on IHC [14]. 

The differential diagnoses of IMT include nodular fasciitis, sarcomatoid carcinoma, desmoid-type fibromatosis, inflammatory leiomyosarcoma, inflammatory fibroid polyp, and gastrointestinal stromal tumor (GIST), and these are mostly resolved by a combination of distinctive histological features and their negative staining with ALK1 IHC [1,8].

Surgical excision is the preferred treatment of IMTs, despite a recurrence rate of 25% following excision [8]. For prostatic tumors, radical total prostatectomy is increasingly being shown to be an effective treatment strategy, with the fewest cases of recurrence compared to other surgical modalities, such as TURP [16]. Our patient underwent simple (open) prostatectomy because of his peculiar presentation, which necessitated prioritizing symptomatic relief and our inability to make the diagnosis of IMT via needle biopsy prior to surgical intervention. The aforementioned TKI, crizotinib, has been successfully trialed for the treatment of IMT, albeit as second-line therapy for recurrent cases and those not amenable to surgical treatment [14]. Prior genomic profiling of IMTs done by Lovly et al. demonstrated the presence of kinase fusions in most of the tumors, paving the way for the current use of TKIs in the treatment of IMT [5].

## Conclusions

We have presented the case of a 77-year-old man with IMT, presenting with hematuria and acute urine retention, clinically assumed to be BPH. Definitive diagnosis of prostate gland lesions via radiologically guided needle biopsy should be a sine qua non for the management of prostatic pathologies. Diligence in gross specimen examination, an open mind during routine microscopic examination, and the judicious use of IHC are strongly recommended for the pathologist. The use of NGS techniques is to be highly encouraged, especially. Finally, IMT should always be considered in the differential diagnoses of prostatic lesions, even in the elderly, presenting with nonspecific lower urinary tract obstructive symptoms.
